# The Preservation of *PPARγ* Genome Duplicates in Some Teleost Lineages: Insights into Lipid Metabolism and Xenobiotic Exploitation

**DOI:** 10.3390/genes13010107

**Published:** 2022-01-04

**Authors:** Inês Páscoa, Elza Fonseca, Renato Ferraz, André M. Machado, Francisca Conrado, Raquel Ruivo, Isabel Cunha, Luís Filipe C. Castro

**Affiliations:** 1 CIIMAR/CIMAR—Interdisciplinary Centre of Marine and Environmental Research, University of Porto, 4450-208 Porto, Portugal; mipascoa@gmail.com (I.P.); fonseca.ess@gmail.com (E.F.); renatobarbosaferraz@gmail.com (R.F.); andre.machado@ciimar.up.pt (A.M.M.); fi.silva@campus.fct.unl.pt (F.C.); ruivo.raquel@gmail.com (R.R.); 2 Department of Biology, FCUP—Faculty of Sciences, University of Porto, 4169-007 Porto, Portugal

**Keywords:** *PPARγ*, genome duplication, Clupeocephala, sardine, organotins

## Abstract

Three peroxisome proliferator-activated receptor paralogues (*PPARα*, *-β* and *-γ*) are currently recognized in vertebrate genomes. PPARγ is known to modulate nutrition, adipogenesis and immunity in vertebrates. Natural ligands of PPARγ have been proposed; however, the receptor also binds synthetic ligands such as endocrine disruptors. Two paralogues of *PPARα* and *PPARβ* have been documented in teleost species, a consequence of the 3R WGD. Recently, two *PPARγ* paralogue genes were also identified in *Astyanax mexicanus*. We aimed to determine whether the presence of two *PPARγ* paralogues is prevalent in other teleost genomes, through genomic and phylogenetic analysis. Our results showed that besides Characiformes, two *PPARγ* paralogous genes were also identified in other teleost taxa, coinciding with the teleost-specific, whole-genome duplication and with the retention of both genes prior to the separation of the Clupeocephala. To functionally characterize these genes, we used the European sardine (*Sardina pilchardus*) as a model. *PPARγA* and *PPARγB* display a different tissue distribution, despite the similarity of their functional profiles: they are unresponsive to tested fatty acids and other human PPARγ ligands yet yield a transcriptional response in the presence of tributyltin (TBT). This observation puts forward the relevance of comparative analysis to decipher alternative binding architectures and broadens the disruptive potential of man-made chemicals for aquatic species.

## 1. Introduction

Nuclear receptors (NRs) are metazoan-specific transcription factors that modulate the expression of target genes involved in multiple aspects of physiological homeostasis e.g., [1,2]. Among NRs, we find the peroxisome proliferator-activated receptors (*PPARα*, *-β* and *-γ*), which control multiple aspects of lipid metabolism and have been described in vertebrate and invertebrate species [3,4]. *PPARα*, *PPARβ* and *PPARγ* are classical examples of paralogous genes originated from whole-genome duplication (WGD) events that occurred in the vertebrate ancestor. In some invertebrates, such as *Saccoglossus kowalevskii* (Hemichordata) [5], nonvertebrate chordates (amphioxus (Cephalochordata) and sea squirts (Tunicata)) [5,6], and protostome molluscs [4,7], a single *PPAR* orthologous gene is found. On the other hand, in some teleost species, *PPARα* and *PPARβ* genes duplicated during the teleost-specific third round of WGD (3R WGD) event [6,7,8,9,10,11,12]. Intriguingly, in the case of *PPARγ*, a second gene was reported in *Astyanax mexicanus* (Mexican tetra) [13].

In general, *PPAR* paralogues display a characteristic gene expression pattern, modulating different metabolic processes; specifically, *PPARγ* is highly expressed in tissues with active lipid metabolism [14], mostly due to its involvement in the regulation of adipogenesis and lipid storage as well as lipid metabolism and energy homeostasis [15]. In addition to this, other *PPARγ*-related processes have also been described. With respect to teleosts, *PPARγ* has been associated with the regulation of nutrition [16,17], adipogenesis [13,18], immunity [19,20] and was shown to be targeted and disrupted by environmental chemicals [6,16,21].

Proposed endogenous ligands of PPARγ include polyunsaturated fatty acids (PUFA), such as docosahexaenoic acid (DHA), eicosapentaenoic acid (EPA), arachidonic acid (ARA) and linoleic acid (ALA), as well as some of their derivatives, particularly oxygenated metabolites such as oxylipins; in addition, some monounsaturated fatty acids (MUFA) may also act as PPARγ ligands [22,23,24,25,26]. Regarding synthetic ligands, thiazolidinediones that function as insulin sensitizers in humans, and several nonsteroidal anti-inflammatory drugs, may also exert their effect when bound to human PPARγ [26]. Importantly, several environmental contaminants, such as phthalates, perfluorinated compounds and halogenated derivatives of bisphenol A have been shown to activate both human and *Danio rerio* (zebrafish) PPARγ, disrupting lipid homeostasis [27,28,29]. The antifouling tributyltin (TBT), also shown to interfere with lipid metabolism, presents an additional scenario; while serving as partial agonist to most vertebrate PPARγ, it was suggested to exert no activation effect in the teleost PPARγs tested so far (i.e., *Pleuronectes platessa* (European plaice), *D. rerio* (zebrafish), or *Pantodon buchholzi* (butterflyfish) [6,30,31].

In the present work, we provide an exhaustive examination of *PPARγ* gene repertoire in different teleost fish species. Through comparative genomic and phylogenetic analyses, we show that the *PPARγ* gene duplicated in the ancestor of teleosts, and that both paralogues were retained in Salmoniformes, Clupeiformes, Siluriformes, Characiformes and Esociformes taxa. Furthermore, expression profiles and functional characterization of European sardine’s (*Sardina pilchardus*) *PPARγA* and *PPARγB* is provided. Despite the differences in *PPARγA* and *PPARγB* gene expression patterns across several tissues of sardine, similar ligand-binding profiles were found, including activation by TBT, previously unreported within teleosts.

## 2. Materials and Methods

### 2.1. Phylogenic Analysis

A phylogenetic analysis was performed to establish the orthology of novel *PPARγ* sequences. Amino acid sequences from teleosts belonging to several taxonomic orders were collected from GenBank and Ensembl. In addition, *PPARα* and *PPARβ* amino acid sequences of a reptile (*Anolis carolinensis*), an amphibian (*Xenopus tropicalis*), two Actinopterigii (*D. rerio* and *Lepisosteus oculatus*), a bird (*Gallus gallus*) and two mammals (*Homo sapiens* and *Mus musculus*) were used to root the phylogenetic tree. The gathered amino acid sequences were aligned to identify and exclude partial and/or low-quality sequences for further analyses, resulting in a final dataset of 68 PPAR amino acid sequences (accession numbers in Table S1). A MAFFT alignment was generated using MPI Bioinformatics Toolkit [32]. Columns containing 50% gaps were stripped, resulting in a final alignment with 483 positions for phylogenetic analysis. Maximum Likelihood phylogenetic analysis was performed in PhyMl V3.0 [33] using JTT + G + I + F model as the best evolutionary model, as determined by Smart Model Selection (SMS) [34]. The branch support for the phylogenetic tree was calculated using 1000 bootstraps. The resulting tree was visualized in FigTree V1.3.1 (available at http://tree.bio.ed.ac.uk/software/figtree/, accessed on 15 February 2021).

### 2.2. Synteny Analysis

The genomic regions containing Atlantic herring (*Clupea harengus*) *PPARγA* and *PPARγB* genes were localized in the scaffolds NW_012218338.1 (890.5kb) and NW_012221131.1 (264.7kb), respectively. Nine neighboring genes from both sides of these target genes were retrieved from the GenBank database to assemble synteny maps. Atlantic herring loci (GCA_000966335.1) were further used as a reference to BLAST against the genome of the remaining 10 species: European sardine (*S. pilchardus*; GCA_003604335.1), northern pike (*Esox lucius*; GCA_000721915.3), channel catfish (*Ictalurus punctatus*; GCA_001660625.2), Mexican tetra (*A. mexicanus*; GCA_000372685.2), red-bellied piranha (*Pygocentrus nattereri*; GCA_001682695.1), Atlantic salmon (*Salmo salar*; GCA_000233375.4), zebrafish (*D. rerio*; GCA_000002035.4), Amazon molly (*Poecilia formosa*; GCA_000485575.1), barramundi perch (*Lates calcarifer*; GCA_001640805.1) and spotted gar (*L. oculatus*; GCA_000242695.1).

### 2.3. Relative Gene Expression Analysis in the European Sardine

The genome assembly and RNA-Seq datasets of the European sardine [35] were used to determine the relative gene expression (RGE) in various tissues. The *S. pilchardus* genome (SP_G) assembly and 11 RNA-Seq datasets (spleen, midgut, red muscle, kidney, liver, gonad, head kidney, gill + branchial arch, caudal fin (skin + cartilage + bone), brain + pituitary gland and white muscle) were downloaded from NCBI, while the .gtf annotation file was retrieved from ORCAE (https://bioinformatics.psb.ugent.be/gdb/Spil/, accessed on 15 February 2021). The RGE was accessed using a reference-based approach with Hisat2 V.2.2.0 [36] and StringTie V.2.1.2 [37] software. Briefly, the *PPARγA* (Spil_000003g0143.1) and *PPARγB* (Spil_000027g0044.1) genes were identified via blast in the ORCAE database (https://bioinformatics.psb.ugent.be/orcae/overview/Spil, accessed on 22 February 2021). Next, Hisat2 was used to align the RNA-Seq datasets to the genome, and StringTie was used to perform gene abundance quantification, under the defaults, following the protocol of [38]. Gene abundance was quantified in transcript per million (TPM). TPM values were them Log 2-transformed after adding a value of one (log2 (TPM+1)).

### 2.4. Isolation and Cloning of PPARγ Gene Paralogues of the European Sardine

Liver tissue from a single specimen of European sardine was collected and preserved in RNAlater at −20 °C. Total RNA was then extracted using Illustra RNAspin Mini RNA Isolation Kit (GE Healthcare, Chicago, IL, USA), following manufacturer’s instructions. Isolated RNA was treated with RNase-free DNase I and eluted with RNase-free water. After RNA quality and quantity assessment using a microplate spectrophotometer (Take 3 and Synergy HT Multi-Mode Microplate Reader, Biotek, Agilent, Santa Clara, CA, USA), 500 ng of liver RNA was used for cDNA synthesis with iScript^TM^ cDNA Synthesis Kit (Bio-Rad, Hercules, CA, USA), considering the manufacturer’s recommendations.

Two gene sequences of *PPARγ-like* were identified in a liver transcriptome dataset of *S. pilchardus* [39]. Two sets of specific primers, including regions for XbaI and KpnI restriction enzymes, were designed to isolate the ligand binding domain (LBD) of both paralogous genes in sardine. Phusion Flash PCR Master Mix (Thermo Fisher Scientific, Waltham, MA, USA) and specific primer pairs (PPARγA Fw—5’ TTAATCTAGATTGGCGGAGGTCTCGG 3’ and PPARγA Rv—5’ GAGCGGTACCCTAGTACAAGTCTCTGATGATC 3’; PPARγB Fw—5’ AAATTCTAGACTGGGTGAAATTGCCAGG 3’ and PPARγB Rv—5’ AAATGGTACCCTAGTAGAGGTCCCTCATGA 3’) were then used in a polymerase chain reaction (PCR) that comprised an initial step at 98 °C for 10 s followed by 40 cycles at 98 °C for 1 s, 58 °C (PPARγA) or 56 °C (PPARγB) for 5 s, and 72 °C for 15 s, with a final extension step for 60 s. At the end, PCR products were loaded onto a 2% agarose gel, stained with GelRed, and those corresponding to the predicted size were cut and purified with NZYGelpure kit (NZYTech, Lisbon, Portugal).

PCR products and the cloning vector pBIND (AF264722; Promega, Madison, WI, USA) were then digested with XbaI and KpnI (NZYTech) and ligated with T4 ligase (Promega) to produce two GAL4 DBD/PPARγ LBD hybrid proteins, one for each sardine *PPARγ* gene sequence, featuring the DNA binding domain (DBD) of GAL4 and the LBD of one of the *PPARγ* paralogues. The produced hybrid protein will conditionally bind to the upstream activation sequences (UAS) of a second experimental vector containing a luciferase reporter gene (pGL4.31; DQ487213). Besides the hybrid protein, the pBIND vector also expresses *Renilla* luciferase, used to normalize firefly luciferase activity. The plasmid sequences were confirmed by Sanger sequencing (Eurofins Genomics, Ebersberg, Germany).

### 2.5. Cell Culture and In Vitro Transactivation Assays

COS-1 cells were cultured in DMEM with phenol red (PAN-Biotec, Aidenbach, Germany), supplemented with 10% fetal bovine serum (PAN-Biotec) and 1% penicillin/streptomycin (PAN-Biotec), and maintained at 37 °C in a humidified atmosphere with 5% CO_2_.

For luciferase-based reporter transactivation assays, COS-1 cells were seeded 24 h before transfection in 24-well culture plates at a density of 2 × 10^5^ live cells/mL in DMEM with phenol red supplemented as described before. The next day, cells were transfected with 500 ng of pBIND and 1000 ng of pGL4.31, using Lipofectamine 2000 reagent (Thermo Fisher Scientific), in Opti-MEM reduced-serum medium (Gibco, Carlsbad, CA, USA), following the manufacturer’s instructions. After 5 h of incubation, transfection medium was removed and cells were exposed to 1 mL of DMEM without phenol red (PAN-Biotec), previously supplemented with 10% of charcoal-treated fetal bovine serum (PAN-Biotec) and 1% of penicillin/streptomycin, containing 1 µL of each test compound (explained in detail below). Dimethyl sulfoxide (DMSO; Sigma-Aldrich, St. Louis, MO, USA) was used as solvent control, at a final concentration of 0.1% per well. After 24 h of exposure, cells were lysed and firefly luciferase (pGL4.31) and *Renilla* luciferase (pBIND) activities were determined using a Dual Luciferase Assay System kit (Promega, Madison, WI, USA), considering manufacturer’s recommendations, in a Synergy HT Multi-Mode Microplate Reader. 

All assays were repeated three times independently, with two technical replicates per condition being performed each time. Beyond the European sardine PPARγA and PPARγB, these assays were also performed with human PPARγ (kindly provided by Ana Capitão) for comparison purposes, and to guarantee the reliability of the results.

### 2.6. Chemicals and Solutions for the In Vitro Experiments 

COS-1 cells were exposed to three different concentrations of PPARγ agonist tributyltin chloride (TBT: 10, 100 and 250 nM; Sigma-Aldrich, St. Louis, MO, USA); to rosiglitazone (10 µM; Bertin Bioreagent, Montigny le Bretonneux, France) and T0070909 (10 µM; Bertin Bioreagent, Montigny le Bretonneux, France), human PPARγ agonist and antagonist, respectively; and to fatty acids (FA; Sigma-Aldrich, St. Louis, MO, USA), arachidonic acid (ARA, 200 µM), docosahexaenoic acid (DHA, 200 µM) and eicosapentaenoic acid (EPA, 200 µM). Two dilutions (50% and 25%) of a mixture composed of seven FAs (FA mix; 42.9 mM ARA, 42.9 mM EPA, 42.9 mM DHA, 42.9 mM oleic acid, 42.9 mM ALA, 42.9 mM γ-linolenic acid and 11.4 mM palmitic acid) were also tested. All the stock solutions and respective dilutions were prepared in DMSO, with its final concentration being 0.1% in each well.

### 2.7. Statistical Analysis

To determine the statistical significance of the transactivation results, firefly luciferase activity (reported by pGL4.31) was first normalized using *Renilla* luciferase activity (internal control for transfection efficiency expressed by pBIND). Final results were presented as fold-induction variations with respect to the solvent control (DMSO). Normalized values of the different replicates were Ln(Log e)-transformed, and a one-way ANOVA followed by Tukey’s post hoc test were performed, using Sigma Stat (version 11, SPSS Inc.), to compare the results. A *p*-value (*p*) ≤ 0.05 was considered statistically significant. Results are presented as mean ± standard error of the mean (SEM).

### 2.8. Homology Modelling 

The tridimensional models of the LBD of the European sardine PPARγA and PPARγB were deduced using a Swiss-Model homology modelling workspace in alignment mode [40,41]. Human PPARγ crystal structure 3WJ4 was selected as the scaffold template. The quality of the obtained protein structure model was estimated using sequence identity, GMQE (global model quality estimation) and QMEAN (qualitative model energy analysis) [42,43]. Models were visualized, inspected, and aligned to human crystal structure 3WJ4 in PyMOL v1.74 [44].

## 3. Results

### 3.1. Phylogenetic and Synteny Analyses

**Figure 1:**
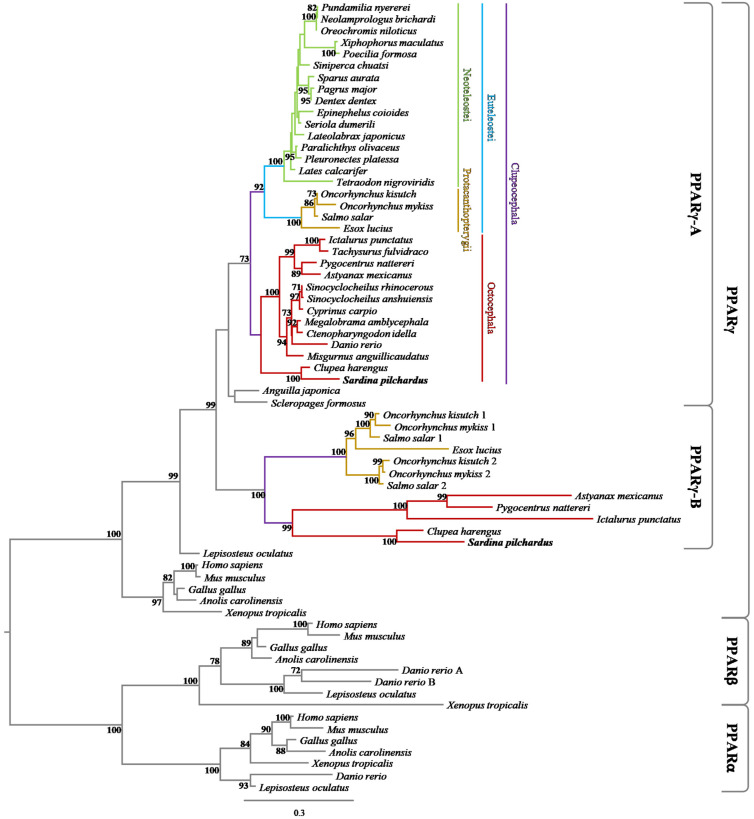
Maximum likelihood phylogenetic analysis of *PPARγA* and *PPARγB* paralogous genes. The numbers at the nodes indicate bootstrap support values.

The Maximum Likelihood phylogenetic analysis (Figure 1) performed with 53 PPARγ sequences, 7 PPARα sequences and 8 PPARβ, revealed two different PPARγ clades: one including mammal, bird, reptile and amphibian PPARγ genes, and the other containing fish PPARγs. In the fish PPARγ group, the non-teleost fish spotted gar (*L. oculatus*) branched off from the teleost PPARγ clade. In agreement with the 3R WGD (third Round of Whole-Genome Duplication), two branches diverged in the teleost PPARγ group, corresponding to the PPARγ gene duplication in the last common ancestor of teleosts. Hence, both *PPARγA* and *PPARγB* paralogous genes are identified in some teleost orders, namely, Salmoniformes (*Oncorhynchus mykiss*, *Oncorhynchus kisutch* and *S. salar*), Clupeiformes (*C. harengus* and *S. pilchardus*), Siluriformes (*I. punctatus*), Characiformes (*A. mexicanus* and *P. nattereri*) and Esociformes (*E. lucius*). Furthermore, duplicate *PPARγB* genes (*PPARγB1* and *PPARγB2*) were observed in the salmonids as result of the salmonid-specific fourth round (4R) of WGD (Figure 1).

**Figure 2:**
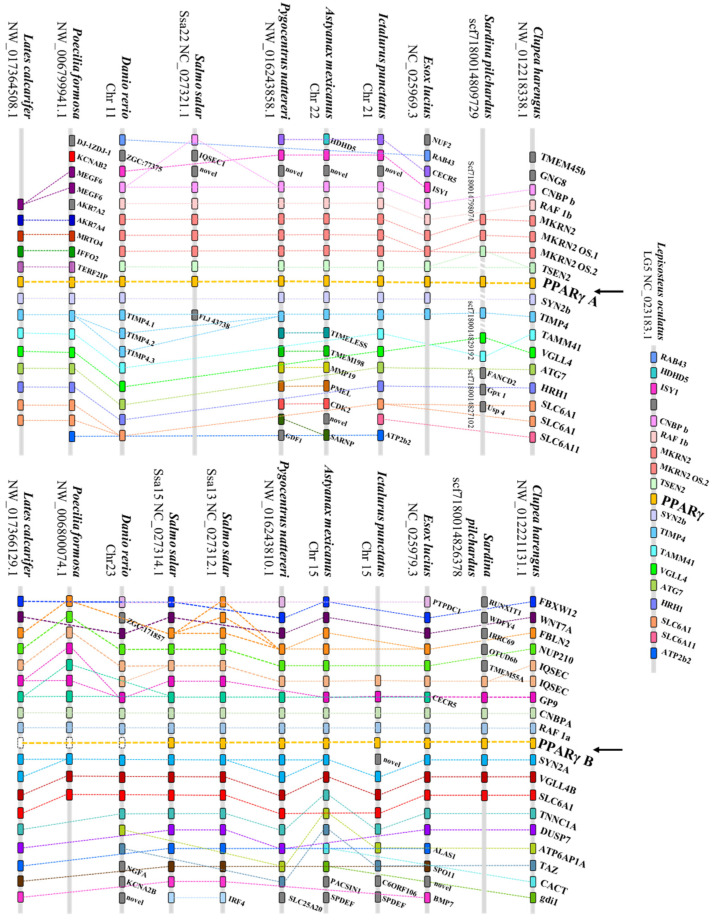
Synteny maps of *PPARγA* and PPARγB loci in representative ray-finned fish species, namely the teleosts Atlantic herring (*Clupea harengus*), common European sardine (*Sardina pilchardus*), northern pike (*Esox lucius*), channel catfish (*Ictalurus punctatus*), Mexican tetra (*Astyanax mexicanus*), red-bellied piranha (*Pygocentrus nattereri*), Atlantic salmon (*Salmo salar*), zebrafish (*Danio rerio*), Amazon molly (*Poecilia formosa*) and barramundi perch (*Lates calcarifer*). Beyond these, the synteny map of the nonteleost spotted gar (*Lepisosteus oculatus*) *PPARγ* loci is also presented. Legend: white box with dashed limit means that gene was lost.

The retention or loss of *PPARγ* paralogous genes was also confirmed by examining *PPARγA* and *PPARγB* loci composition and neighborhood in 10 teleosts and in a non-teleost (*L. oculatus)* fish species (Figure 2). Synteny analysis showed that *PPARγA* and *PPARγB* neighboring genes are mostly conserved, even in teleosts retaining a single *PPARγ* paralogous copy (Figure 2).

### 3.2. Relative Gene Expression Analysis

**Figure 3:**
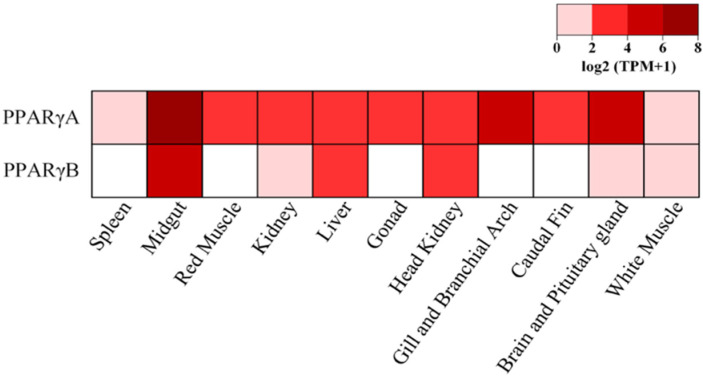
Relative gene expression patterns of common European sardine *PPARγA* and *PPARγB* genes in 11 tissues. Relative gene expression levels are provided as Log 2-transformed transcript per million (TPM) adding a value of one [log 2 (TPM+1)], from 0 to 8.

We next examined the relative gene expression (RGE) of the two *PPARγ* paralogues using the European sardine as a model. RGE analysis was performed using 11 tissues (Figure 3). Our analysis shows that *PPARγA* is ubiquitously expressed in all tested tissues, contrarily to *PPARγB* that showed higher expression in midgut, liver and head kidney, and residual expression in kidney, brain and pituitary gland and white muscle. No *PPARγB* expression was observed in spleen, red muscle, gonad, gills and branchial arches or caudal fin tissues. In general, the comparison of RGE among tissues, especially in kidney and brain and pituitary gland, suggests higher expression levels of *PPARγA* than *PPARγB*. However, in liver, head kidney and white muscle, both paralogues presented similar gene expression (Figure 3 and Table S2).

### 3.3. Transactivation Assays

**Figure 4:**
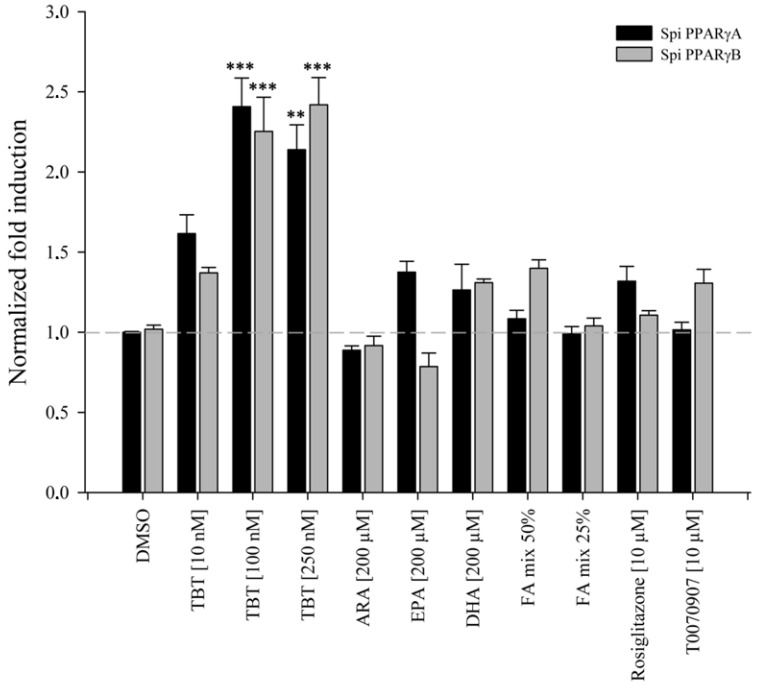
Results of transactivation assays with European sardine PPARγA and PPARγB. In these assays, transiently transfected COS-1 cells were exposed to three concentrations of tributyltin chloride (TBT; 10 nM, 100 nM and 250 nM), two concentrations of a mixture of seven fatty acids (FA mix; 50% and 25%) and one concentration of arachidonic acid (ARA, 200 µM), eicosapentaenoic acid (EPA, 200 µM), docosahexaenoic acid (DHA, 200 µM), rosiglitazone (human PPARγ agonist; 10 µM) and T0070909 (human PPARγ antagonist; 10 µM). Dimethyl sulfoxide (DMSO; 0.1%) was used as solvent control. Data are shown as mean ± standard error of the mean (SEM). Legend: ** and *** shows a statistically significant response of *p* ≤ 0.01 and *p* ≤ 0.001, respectively, when compared to DMSO.

We next examined the ligand binding profile of European sardine PPARγA and PPARγB in the presence of proposed PPARγ ligands (Figure 4). Tributyltin (TBT), a known obesogen shown to bind to several vertebrate PPARγ receptors, excluding teleosts [6,30], significantly activated both sardine PPARγ receptors when compared to the solvent control (DMSO), except at the lowest TBT concentration (10 nM). Regarding polyunsaturated FAs (200 μM) and FAs mixtures (50% and 25% diluted), no significant activation or repression of European sardine PPARγ paralogues was detected when compared to the solvent control. Similarly to FAs, neither rosiglitazone nor T0070909 (10 μM), agonist and antagonist of the human PPARγ, respectively, had a significant effect on the European sardine PPARγA or PPARγB modulation (Figure 4) relative to the DMSO. The human PPARγ LBD, used as control, was significantly activated by TBT (100 and 250 nM), FAs (200 μM and 50% and 25% dilution mixes) and rosiglitazone (10 μM), and repressed by T0070909 (10 μM), as previously described (Figure S1).

### 3.4. Sequence Analysis and Homology Modelling 

**Figure 5:**
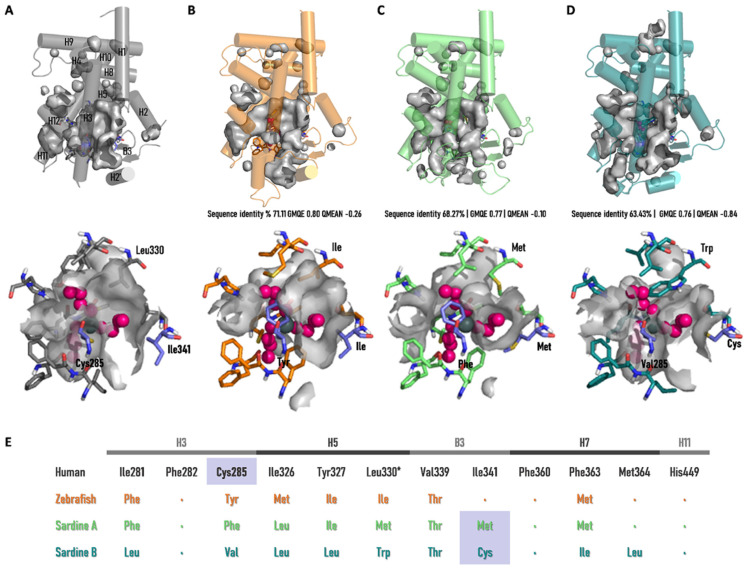
PPARγ ligand-binding pocket (LBP) cavities analysis. (**A**) Human PPARγ ligand-binding domain (LBD) crystal structure 3WJ4 with representation of LBD cavities (top), and detailed view of the LBP cavity with the residues that interact with TBT (Harada et al. 2015 [45]) depicted as sticks (down). (**B–D**) Zebrafish PPARγ, sardine PPARγA and sardine PPARγB homology models with representation of LBD cavities (top), and detailed view of the LBP cavities with the corresponding interacting residues depicted as sticks (down). TBT molecule from crystal structure 3WJ4 is shown in magenta. Sequence identity (%), global model quality estimation (GMQE), QMean are indicated below each model. (**E**) Human PPARγ residues that interact with TBT and the corresponding residues found in Zebrafish PPARγ, sardine PPARγA and sardine PPARγB. The location of the residues in the structure is indicated (H, helix; B, β-strand). * Indicates a residue substitution in sardine PPARγB, resulting in an aromatic ring protrusion.

To further address the TBT activation profile, previously unreported in teleost fish, we deduced European sardine PPARγA and PPARγB tridimensional structures and examined the conservation of LBD residues reported to interact with TBT, through structural and sequence alignment, using the human PPARγ crystal structure (3WJ4) as a template (Figure 5). Overall, the TBT-interacting amino acid composition of the teleost sequences are poorly conserved, as previously reported for *D. rerio* (zebrafish) and *P. buchholzi* (butterflyfish) [6]. The observed substitutions occur between amino acids with hydrophobic side chains, yet an increase in aromatic ring content is also noted, with smaller hydrophobic amino acids replaced by residues with aromatic hydrophobic side chains (Phe, Trp, or Tyr). While these substitutions maintain the hydrophobic core of the binding pocket, they impose distinct spatial and volume constraints within the binding cavity. For instance, as previously noted, Cys285Tyr replacement in zebrafish and butterflyfish leads to an aromatic ring protrusion into the pocket [6], similar to the Cys285Phe and Leu330Trp substitutions observed in European sardine PPARγA and PPARγB, respectively. More specifically, we examined the conservation status of a signature cysteine in helix 3 (Cys285, H3), with a sulfur-containing side chain shown to bind to the tin atom of TBT [45]. Curiously, this residue, critical for TBT-induced activation in other non-teleost species, is not conserved in the TBT-responsive European sardine PPARγA and PPARγB [6]. Despite the absence of a Cys residue in H3, additional sulfur-containing amino acids are overserved in the β-strand B3 of European sardine PPARγA and PPARγB (Met and Cys, respectively), but not in zebrafish. In the human PPARγ, TBT partial agonist activity was suggested to derive predominantly from interactions with residues in H3 and B3 [45].

## 4. Discussion

During the evolution of vertebrates, polyploidization events have been proposed to provide the new genetic material for the emergence of novelties [46,47,48,49,50,51]. Although the exact duplication timings have fueled intense debate, the current consensus suggests one shared genome duplication (1R; first Round) between cyclostomes and gnathostomes, with independent duplication taking place in each lineage independently [52]. In teleosts, a 3R WGD (Whole-Genome Duplication) is proposed at the origin of teleosts [53]. Besides this last WGD, a 4R WGD was inferred in salmonids [53,54,55,56]. In the context of NR (Nuclear Receptor) gene evolution, WGD occurrences have been central components. Recently, Capitão and collaborators [6] suggested that *PPAR-like* sequences present in all vertebrate lineages result from WGD events that occurred before gnathostome radiation and after tunicate divergence, yielding the three *PPAR* paralogous genes. The consequences of the 3R WGD on PPAR gene numbers has also been previously established for both *PPARα* and *PPARβ* genes [8,11,57,58,59,60,61]. Recently, Wafer and collaborators [13] identified two *PPARγ* paralogous genes in Mexican tetra (*A. mexicanus)*, suggesting a pronounced loss of duplicated *PPARγ* in other teleost lineages. In the present work, by means of comparative genomics and phylogenetic analyses, we investigate the presence of *PPARγ* paralogous genes in an ample collection of ray-finned fish lineages. As expected, spotted gar (*L. oculatus*) presented a single *PPARγ* copy, since this species diverged prior to the 3R WGD event (Figure 1 and Figure 2). Moreover, our analyses showed that two *PPARγ* paralogous genes (Figure 2) were retained not only in Characiformes (tetras and piranhas), but also in Salmoniformes (salmons and trouts), Clupeiformes (herrings and sardines), Siluriformes (catfishes), and Esociformes (pikes) taxa (Figure 1). These observations suggest a wider retention of *PPARγ* paralogues than previously anticipated [13]. Additional salmonid-specific *PPARγ*B duplicates were found in Atlantic salmon (*S. salar*), coho salmon (*O. kisutch*) and rainbow trout (*O. mykiss*), which resulted of the 4R WGD. The presence of both *PPARγ* paralogues in such lineages and the retention of a single *PPARγ* paralogue in the early-diverging teleost lineages such as Elopomorpha (e.g., eels) and Osteoglossomorpha (e.g., butterflyfishes), allows us to estimate that the retention of *PPARγ* duplicated genes occurred at the time of Clupeocephala divergence, approximately 185-190 Million years ago [62]. Thus, similarly to *PPARα* and *PPARβ*, *PPARγ* duplication resulted from 3R WGD, with episodes of gene loss shaping teleost *PPARγ* gene repertoire.

Human PPARs can be activated by both saturated and unsaturated dietary FAs, as well as lipids involved in intracellular signaling pathways [63,64,65]. When binding to these ligands, PPARs play an important physiological role, with PPARγ being crucial for lipid storage and adipogenesis [66]. To determine the binding profile of European sardine’s PPARγA and PPARγB, we assessed the ability of transiently transfected mammalian cells to induce reporter gene transcription upon exposure to ligands able to bind and modulate human PPARγ (see Figure S1). Regarding FAs, no significant responses were observed with the European sardine *PPARγ* paralogues. This agrees with previous reports with plaice (*P. platessa*) and gilthead sea bream (*Sparus aurata*) PPARγs, also yielding no activation upon exposure to FAs [57]. In the chondrichthyan *Leucoraja erinacea* (little skate) on the other hand, FAs were shown to be potent inducers of PPARγ [6]. These disparate observations could be due to structural constraints in the binding cavity of teleost PPARγs. In fact, the deduced tridimensional structures of the European sardine, zebrafish and butterflyfish PPARγs show low residue conservation, displaying more amino acid residues with bulky aromatic side chains possibly affecting pocket size and architecture, whereas the little skate binding pocket is highly conserved when compared to human PPARγ [6]. Null activation profiles were obtained with the synthetic PPARγ agonist and antagonists, in agreement with previous reports on the modulation of zebrafish PPARγ by rosiglitazone [27]. Sardine PPARγ paralogues were also tested for their susceptibility towards TBT (tributyltin) binding. TBT is a currently restricted organotin compound, long used as an antifouling agent in general maritime industries [67,68,69]. In aquatic environments, exposure to TBT mediates endocrine disruption, causing an abnormal induction of male sex characters in females in gastropod mollusks [70,71] and fishes [72,73]. TBT is also considered an obesogen since it is able to bind and greatly induce PPARγ activity, disturbing lipid and energy metabolism in some vertebrates [6,74,75]. Crystallographic analysis of human PPARγ bound to TBT unraveled a partial agonist binding mode, with the ligand TBT interacting with a sulfur-containing cysteine residue (Cys285) on helix 3 (H3) and establishing hydrophobic interactions with the side chains of two residues (Val339 and Ile341) embedded on the β-strand (B3), inducing conformational changes that allow cofactor binding and transcription [45]. Cysteine residues have been shown to be crucial for the anchorage of TBT, not only in PPARs but also in the Retinoid X Receptor (RXR), through interaction of the tin atom with the highly reactive sulfhydryl group of the cysteine side chain [45,76]. In agreement, teleost PPARγs lacking such signature cysteine (e.g., plaice, zebrafish or butterflyfish) were shown to be unresponsive towards growing concentrations of TBT [6,30]. Furthermore, the substitution of the cysteine residue in human and little skate PPARγs abolished TBT-mediated reporter gene transcription in vitro [6,45]. Yet, both European sardine PPARγA and PPARγB were able to induce reporter gene transcription upon TBT exposure, despite lacking the conserved cysteine in H3 (Figure 4 and Figure 5). This observation challenges the universality of the TBT-dependent PPARγ activation mechanism. Careful analysis of pocket residue composition highlights possible alternatives. In fact, sardine PPARγB exhibits an available cysteine residue in B3 to anchor TBT, whereas PPARγA has a methionine. Similarly to cysteine, methionine is a sulfur-containing amino acid, and despite its lower reactivity could also react with the tin atom [77]. In spite of the consensus on the crucial role of cysteine for TBT binding, unusual binding modes have been previously observed. For instance, in the Echinodermata *Paracentrotus lividus*, PPARγ is unresponsive to TBT despite the conservation of H3 cysteine, whereas the cysteine-to-tyrosine substitutions observed in teleosts such as zebrafish and in the mollusk *Patella depressa* yield null or repressive responses, respectively [4,5,6]. Alternative modes for TBT binding have also been proposed for RXR [78]. Thus, future structural studies should address these potential alternative binding architectures.

Although no differences were found in the ligand-binding profile of sardine PPARγA and PPARγB, relative gene expression analysis shows a distinct tissue distribution pattern, with the European sardine *PPARγA* displaying ubiquitous expression in all analyzed tissues, whereas *PPARγB* is only expressed in midgut, kidney, liver, head kidney, brain and pituitary gland, and white muscle (Figure 3 and Table S2). In mammals, *PPARγ* expression is detected in the gastrointestinal tract (only during embryonic development), liver, kidney, heart, adipose tissues, skeletal muscle, placenta and lung. Further promoter usage and alternative splicing lead to the differential expression of PPARγ splice variants in each of these tissues reviewed in [3,79,80]. Thus, the retention of both PPARγ genes in various teleost lineages could be related, with a distinct spatial and temporal distribution.

## 5. Conclusions

In conclusion, this study indicates that 3R WGD led to *PPARγ* gene duplication, as previously described for *PPARα* and *PPARβ* paralogues. *PPARγ* paralogue retention was first reported in *A. mexicanus* (Characiformes), however, we suggest that PPARγ paralogue retention affected many other teleost lineages. In spite of the differential tissue distributions and expression patterns of sardine *PPAR*γA and *PPAR*γB, indicating tissue-specific functions, both *PPAR*γ genes presented similar ligand preferences. Nevertheless, the modulation of sardine *PPAR*γ paralogues by TBT challenges current paradigms and emphasizes the need to further address the mechanisms of ligand-binding and the susceptibility of teleost lineages towards organotin exposure.

## Data Availability

Data available as supplementary material. Other can be requested from the corresponding authors.

## Supplementary Materials

The following are available online at https://www.mdpi.com/article/10.3390/genes13010107/s1, Figure S1: In vitro transcriptional activation of Homo sapiens PPARγ, Table S1: Accession numbers of the sequences used to perform the phylogenic analysis, Table S2: Relative gene expression levels of *PPARγA* and *PPARγB* in *Sardina pilchardus*.

## References

[1] Glass C.K., Rosenfeld M.G. (2000). The coregulator exchange in transcriptional functions of nuclear receptors. Genes Dev..

[2] Bookout A.L., Jeong Y., Downes M., Yu R.T., Evans R.M., Mangelsdorf D.J. (2006). Anatomical Profiling of Nuclear Receptor Expression Reveals a Hierarchical Transcriptional Network. Cell.

[3] Abbott B.D. (2009). Review of the expression of peroxisome proliferator-activated receptors alpha (PPARα), beta (PPARβ), and gamma (PPARγ) in rodent and human development. Reprod. Toxicol..

[4] Capitão A.M.F., Lopes-Marques M., Páscoa I., Sainath S.B., Hiromori Y., Matsumaru D., Nakanishi T., Ruivo R., Santos M.M., Castro L.F.C. (2021). An ancestral nuclear receptor couple, PPAR-RXR, is exploited by organotins. Sci. Total Environ..

[5] Capitão A., Lopes-Marques M., Páscoa I., Ruivo R., Mendiratta N., Fonseca E., Castro L.F.C., Santos M.M. (2020). The Echinodermata PPAR: Functional characterization and exploitation by the model lipid homeostasis regulator tributyltin. Environ. Pollut..

[6] Capitão A.M.F., Lopes-Marques M.S., Ishii Y., Ruivo R., Fonseca E.S.S., Páscoa I., Jorge R.P., Barbosa M., Hiromori Y., Miyagi T. (2018). Evolutionary Exploitation of Vertebrate Peroxisome Proliferator-Activated Receptor γ by Organotins. Environ. Sci. Technol..

[7] Ran Z., Kong F., Liao K., Xu J., Liu X., Shi P., Zhang M., Wu K., Yan X. (2021). Identification and Expression of PPAR in Sinonovacula constricta and Their Potential Regulatory Effects on Δ6 Fad Transcription. J. Ocean Univ. China.

[8] Bertrand S., Brunet F.G., Escriva H., Parmentier G., Laudet V., Robinson-Rechavi M. (2004). Evolutionary Genomics of Nuclear Receptors: From Twenty-Five Ancestral Genes to Derived Endocrine Systems. Mol. Biol. Evol..

[9] Bertrand S., Thisse B., Tavares R., Sachs L., Chaumot A., Bardet P.-L., Escriva H., Duffraisse M., Marchand O., Safi R. (2007). Unexpected Novel Relational Links Uncovered by Extensive Developmental Profiling of Nuclear Receptor Expression. PLoS Genet..

[10] Leaver M.J., Ezaz M.T., Fontagné-Dicharry S., Tocher D.R., Boukouvala E., Krey G. (2007). Multiple peroxisome proliferator-activated receptor β subtypes from Atlantic salmon (Salmo salar). J. Mol. Endocrinol..

[11] Madureira T.V., Pinheiro I., Freire R.P., Rocha E., Castro L.F., Urbatzka R. (2017). Genome specific PPARαB duplicates in salmonids and insights into estrogenic regulation in brown trout. Comp. Biochem. Physiol. Part B Biochem. Mol. Biol..

[12] Schaaf M.J.M. (2017). Nuclear receptor research in zebrafish. J. Mol. Endocrinol..

[13] Wafer R., Tandon P., Minchin J.E.N. (2017). The Role of Peroxisome Proliferator-Activated Receptor Gamma (PPARG) in Adipogenesis: Applying Knowledge from the Fish Aquaculture Industry to Biomedical Research. Front. Endocrinol..

[14] Vidal-Puig A.J., Considine R.V., Jimenez-Liñan M., Werman A., Pories W.J., Caro J.F., Flier J.S. (1997). Peroxisome proliferator-activated receptor gene expression in human tissues. Effects of obesity, weight loss, and regulation by insulin and glucocorticoids. J. Clin. Investig..

[15] Tontonoz P., Hu E., Spiegelman B.M. (1994). Stimulation of adipogenesis in fibroblasts by PPARγ2, a lipid-activated transcription factor. Cell.

[16] Den Broeder M.J., Moester M.J.B., Kamstra J.H., Cenijn P.H., Davidoiu V., Kamminga L.M., Ariese F., De Boer J.F., Legler J. (2017). Altered Adipogenesis in Zebrafish Larvae Following High Fat Diet and Chemical Exposure Is Visualised by Stimulated Raman Scattering Microscopy. Int. J. Mol. Sci..

[17] Wang A., Yang W., Liu F., Yin X., Yu Y. (2017). Peroxisome proliferator-activated receptor gamma (Pparγ) in Redlip Mullet Liza haematocheila: Molecular cloning, tissue distribution, and response to dietary lipid levels. Turk. J. Fish Aquat. Sc..

[18] Den Broeder M.J., Kopylova V.A., Kamminga L.M., Legler J. (2015). Zebrafish as a Model to Study the Role of Peroxisome Proliferating-Activated Receptors in Adipogenesis and Obesity. PPAR Res..

[19] Sundvold H., Ruyter B., Østbye T.-K., Moen T. (2010). Identification of a novel allele of peroxisome proliferator-activated receptor gamma (PPARG) and its association with resistance to Aeromonas salmonicida in Atlantic salmon (Salmo salar). Fish Shellfish. Immunol..

[20] Antonopoulou E., Kaitetzidou E., Castellana B., Panteli N., Kyriakis D., Vraskou Y., Planas J. (2017). In Vivo Effects of Lipopolysaccharide on Peroxisome Proliferator-Activated Receptor Expression in Juvenile Gilthead Seabream (Sparus Aurata). Biology.

[21] Adeogun A.O., Ibor O.R., Regoli F., Arukwe A. (2016). Peroxisome proliferator-activated receptors and biotransformation responses in relation to condition factor and contaminant burden in tilapia species from Ogun River, Nigeria. Comp. Biochem. Physiol. Part C Toxicol. Pharmacol..

[22] Forman B.M., Chen J., Evans R.M. (1997). Hypolipidemic drugs, polyunsaturated fatty acids, and eicosanoids are ligands for peroxisome proliferator-activated receptors and. Proc. Natl. Acad. Sci. USA.

[23] Schopfer F.J., Lin Y., Baker P.R.S., Cui T., Garcia-Barrio M., Zhang J., Chen K., Chen Y.E., Freeman B.A. (2005). Nitrolinoleic acid: An endogenous peroxisome proliferator-activated receptor ligand. Proc. Natl. Acad. Sci. USA.

[24] Liberato M.V., Nascimento A.S., Ayers S.D., Lin J.Z., Cvoro A., Silveira R.L., Martínez L., Souza P.C.T., Saidemberg D., Deng T. (2012). Medium Chain Fatty Acids Are Selective Peroxisome Proliferator Activated Receptor (PPAR) γ Activators and Pan-PPAR Partial Agonists. PLoS ONE.

[25] Yang Z.-H., Miyahara H., Iwasaki Y., Takeo J., Katayama M. (2013). Dietary supplementation with long-chain monounsaturated fatty acids attenuates obesity-related metabolic dysfunction and increases expression of PPAR gamma in adipose tissue in type 2 diabetic KK-Ay mice. Nutr. Metab..

[26] Grygiel-Górniak B. (2014). Peroxisome proliferator-activated receptors and their ligands: Nutritional and clinical implications—a review. Nutr. J..

[27] Riu A., Mccollum C.W., Pinto C.L., Grimaldi M., Hillenweck A., Perdu E., Zalko D., Bernard L., Laudet V., Balaguer P. (2014). Halogenated Bisphenol-A Analogs Act as Obesogens in Zebrafish Larvae (Danio rerio). Toxicol. Sci..

[28] Grimaldi M., Boulahtouf A., Delfosse V., Thouennon E., Bourguet W., Balaguer P. (2015). Reporter cell lines to evaluate the selectivity of chemicals for human and zebrafish estrogen and peroxysome proliferator activated Î³ receptors. Front. Neurosci..

[29] Huang Q., Chen Q. (2017). Mediating Roles of PPARs in the Effects of Environmental Chemicals on Sex Steroids. PPAR Res..

[30] Colliar L., Sturm A., Leaver M.J. (2011). Tributyltin is a potent inhibitor of piscine peroxisome proliferator-activated receptor α and β. Comp. Biochem. Physiol. Part C Toxicol. Pharmacol..

[31] Janesick A., Blumberg B. (2011). Minireview: PPARγ as the target of obesogens. J. Steroid Biochem. Mol. Biol..

[32] Zimmermann L., Stephens A., Nam S.-Z., Rau D., Kübler J., Lozajic M., Gabler F., Söding J., Lupas A.N., Alva V. (2018). A Completely Reimplemented MPI Bioinformatics Toolkit with a New HHpred Server at its Core. J. Mol. Biol..

[33] Guindon S., Dufayard J.-F., Lefort V., Anisimova M., Hordijk W., Gascuel O. (2010). New Algorithms and Methods to Estimate Maximum-Likelihood Phylogenies: Assessing the Performance of PhyML 3.0. Syst. Biol..

[34] Lefort V., Longueville J.-E., Gascuel O. (2017). SMS: Smart Model Selection in PhyML. Mol. Biol. Evol..

[35] Louro B., De Moro G., Garcia C., Cox C.J., Veríssimo A., Sabatino S.J., Santos A.M., Canário A.V.M. (2019). A haplotype-resolved draft genome of the European sardine (Sardina pilchardus). GigaScience.

[36] Kim D., Langmead B., Salzberg S.L. (2015). HISAT: A fast spliced aligner with low memory requirements. Nat. Methods.

[37] Pertea M., Pertea G.M., Antonescu C.M., Chang T.-C., Mendell J.T., Salzberg S.L. (2015). StringTie enables improved reconstruction of a transcriptome from RNA-seq reads. Nat. Biotechnol..

[38] Pertea M., Kim D., Pertea G.M., Leek J.T., Salzberg S.L. (2016). Transcript-level expression analysis of RNA-seq experiments with HISAT, StringTie and Ballgown. Nat. Protoc..

[39] Machado A.M., Tørresen O.K., Kabeya N., Couto A., Petersen B., Felício M., Campos P.F., Fonseca E., Bandarra N., Lopes-Marques M. (2018). “Out of the Can”: A Draft Genome Assembly, Liver Transcriptome, and Nutrigenomics of the European Sardine, Sardina pilchardus. Genes.

[40] Arnold K., Bordoli L., Kopp J., Schwede T. (2006). The SWISS-MODEL workspace: A web-based environment for protein structure homology modelling. Bioinformatics.

[41] Biasini M., Bienert S., Waterhouse A., Arnold K., Studer G., Schmidt T., Kiefer F., Cassarino T.G., Bertoni M., Bordoli L. (2014). SWISS-MODEL: Modelling protein tertiary and quaternary structure using evolutionary information. Nucleic Acids Res..

[42] Benkert P., Tosatto S.C.E., Schomburg D. (2008). QMEAN: A comprehensive scoring function for model quality assessment. Proteins Struct. Funct. Bioinform..

[43] Benkert P., Biasini M., Schwede T. (2011). Toward the estimation of the absolute quality of individual protein structure models. Bioinformatics.

[44] Schrödinger L.L.C. (2010). The PyMOL Molecular Graphics System, Version 1.3.

[45] Harada S., Hiromori Y., Nakamura S., Kawahara K., Fukakusa S., Maruno T., Noda M., Uchiyama S., Fukui K., Nishikawa J.-I. (2015). Structural basis for PPARγ transactivation by endocrine-disrupting organotin compounds. Sci. Rep..

[46] Ohno S. (1970). Evolution by Gene Duplication.

[47] Panopoulou G., Hennig S., Groth D., Krause A., Poustka A.J., Herwig R., Vingron M., Lehrach H. (2003). New Evidence for Genome-Wide Duplications at the Origin of Vertebrates Using an Amphioxus Gene Set and Completed Animal Genomes. Genome Res..

[48] Marlétaz F., Firbas P.N., Maeso I., Tena J.J., Bogdanovic O., Perry M., Wyatt C.D.R., de la Calle-Mustienes E., Bertrand S., Burguera D. (2018). Amphioxus functional genomics and the origins of vertebrate gene regulation. Nature.

[49] Ravi V., Venkatesh B. (2018). The Divergent Genomes of Teleosts. Annu. Rev. Anim. Biosci..

[50] Dehal P., Boore J.L. (2005). Two Rounds of Whole Genome Duplication in the Ancestral Vertebrate. PLoS Biol..

[51] Putnam N.H., Butts T., Ferrier D.E.K., Furlong R.F., Hellsten U., Kawashima T., Robinson-Rechavi M., Shoguchi E., Terry A., Yu J.-K. (2008). The amphioxus genome and the evolution of the chordate karyotype. Nature.

[52] Nakatani Y., Shingate P., Ravi V., Pillai N.E., Prasad A., McLysaght A., Venkatesh B. (2021). Reconstruction of proto-vertebrate, proto-cyclostome and proto-gnathostome genomes provides new insights into early vertebrate evolution. Nat. Commun..

[53] Jaillon O., Aury J.-M., Brunet F., Petit J.-L., Stange-Thomann N., Mauceli E., Bouneau L., Fischer C., Ozouf-Costaz C., Bernot A. (2004). Genome duplication in the teleost fish Tetraodon nigroviridis reveals the early vertebrate proto-karyotype. Nature.

[54] Berthelot C., Brunet F., Chalopin D., Juanchich A., Bernard M., Noel B., Bento P., DA Silva C., Labadie K., Alberti A. (2014). The rainbow trout genome provides novel insights into evolution after whole-genome duplication in vertebrates. Nat. Commun..

[55] Rondeau E.B., Minkley D.R., Leong J.S., Messmer A.M., Jantzen J.R., Von Schalburg K.R., Lemon C., Bird N.H., Koop B.F. (2014). The Genome and Linkage Map of the Northern Pike (*Esox lucius*): Conserved Synteny Revealed between the Salmonid Sister Group and the Neoteleostei. PLoS ONE.

[56] Lien S., Koop B.F., Sandve S.R., Miller J.R., Kent M.P., Nome T., Hvidsten T.R., Leong J.S., Minkley D.R., Zimin A. (2016). The Atlantic salmon genome provides insights into rediploidization. Nature.

[57] Leaver M.J., Boukouvala E., Antonopoulou E., Diez A., Favre-Krey L., Ezaz M.T., Bautista J.M., Tocher D.R., Krey G. (2005). Three Peroxisome Proliferator-Activated Receptor Isotypes from Each of Two Species of Marine Fish. Endocrinology.

[58] Braasch I., Postlethwait J.H., Soltis P.S., Soltis D.E. (2012). Polyploidy in fish and the teleost genome duplication. Polyploidy and Genome Evolution.

[59] Urbatzka R., Galante-Oliveira S., Rocha E., Castro L.F.C., Cunha I. (2013). Tissue expression of PPAR-alpha isoforms in Scophthalmus maximus and transcriptional response of target genes in the heart after exposure to WY-14643. Fish Physiol. Biochem..

[60] Urbatzka R., Galante-Oliveira S., Rocha E., Lobo-Da-Cunha A., Castro L.F.C., Cunha I. (2015). Effects of the PPARα agonist WY-14,643 on plasma lipids, enzymatic activities and mRNA expression of lipid metabolism genes in a marine flatfish, *Scophthalmus maximus*. Aquat. Toxicol..

[61] Cunha I., Galante-Oliveira S., Rocha E., Urbatzka R., Castro L.F.C. (2015). Expression of intercellular lipid transport and cholesterol metabolism genes in eggs and early larvae stages of turbot, *Scophthalmus maximus*, a marine aquaculture species. Mar. Biol..

[62] Hughes L.C., Ortí G., Huang Y., Sun Y., Baldwin C.C., Thompson A.W., Arcila D., Betancur R.-R., Li C., Becker L. (2018). Comprehensive phylogeny of ray-finned fishes (*Actinopterygii*) based on transcriptomic and genomic data. Proc. Natl. Acad. Sci. USA.

[63] Keller H., Dreyer C., Medin J., Mahfoudi A., Ozato K., Wahli W. (1993). Fatty acids and retinoids control lipid metabolism through activation of peroxisome proliferator-activated receptor-retinoid X receptor heterodimers. Proc. Natl. Acad. Sci. USA.

[64] Kliewer S.A., Sundseth S.S., Jones S.A., Brown P.J., Wisely G.B., Koble C.S., Devchand P., Wahli W., Willson T.M., Lenhard J.M. (1997). Fatty acids and eicosanoids regulate gene expression through direct interactions with peroxisome proliferator-activated receptors α and γ. Proc. Natl. Acad. Sci. USA.

[65] O’Sullivan S.E. (2016). An update on PPAR activation by cannabinoids. Br. J. Pharmacol..

[66] Hummasti S., Tontonoz P. (2006). The Peroxisome Proliferator-Activated Receptor N-Terminal Domain Controls Isotype-Selective Gene Expression and Adipogenesis. Mol. Endocrinol..

[67] International Maritime Organization (IMO) (2002). Anti-Fouling Systems. https://www.imo.org/en/OurWork/Environment/Pages/Anti-fouling.aspx.

[68] Appel K.E. (2004). Organotin Compounds: Toxicokinetic Aspects. Drug Metab. Rev..

[69] Okoro H.K., Fatoki O.S., Adekola F.A., Ximba B.J., Snyman R.G., Opeolu B. (2011). Human Exposure, Biomarkers, and Fate of Organotins in the Environment. Rev. Environ. Contam. Toxicol. Vol..

[70] Matthiessen P., Gibbs P.E. (1998). Critical appraisal of the evidence for tributyltin-mediated endocrine disruption in mollusks. Environ. Toxicol. Chem..

[71] Lima D., Reis-Henriques M.A., Silva R., Santos A.I., Castro L.F.C., Santos M.M. (2011). Tributyltin-induced imposex in marine gastropods involves tissue-specific modulation of the retinoid X receptor. Aquat. Toxicol..

[72] McAllister B.G., Kime D.E. (2003). Early life exposure to environmental levels of the aromatase inhibitor tributyltin causes masculinisation and irreversible sperm damage in zebrafish (*Danio rerio*). Aquat. Toxicol..

[73] Shimasaki Y., Kitano T., Oshima Y., Inoue S., Imada N., Honjo T. (2003). Tributyltin causes masculinization in fish. Environ. Toxicol. Chem..

[74] Grn F., Watanabe H., Zamanian Z., Maeda L., Arima K., Cubacha R., Gardiner D.M., Kanno J., Iguchi T., Blumberg B. (2006). Endocrine-Disrupting Organotin Compounds Are Potent Inducers of Adipogenesis in Vertebrates. Mol. Endocrinol..

[75] Grün F., Blumberg B. (2009). Minireview: The Case for Obesogens. Mol. Endocrinol..

[76] le Maire A., Grimaldi M., Roecklin D., Dagnino S., Vivat-Hannah V., Balaguer P., Bourguet W. (2009). Activation of RXR–PPAR heterodimers by organotin environmental endocrine disruptors. EMBO Rep..

[77] Caubère P., Coutrot P., Trost B.M., Fleming I. (1991). Reduction of sulfur–carbon bonds and of other heteroatoms bonded to tetrahedral carbon. Comprehensive Organic Synthesis.

[78] Fonseca E., Ruivo R., Borges D., Franco J.N., Santos M.M., Castro L.F.C. (2020). Of Retinoids and Organotins: The Evolution of the Retinoid X Receptor in Metazoa. Biomolecules.

[79] Zhu Y., Qi C., Korenberg J.R., Chen X.N., Noya D., Rao M.S., Reddy J.K. (1995). Structural organization of mouse peroxisome proliferator-activated receptor gamma (mPPAR gamma) gene: Alternative promoter use and different splicing yield two mPPAR gamma isoforms. Proc. Natl. Acad. Sci. USA.

[80] Fajas L., Auboeuf D., Raspé E., Schoonjans K., Lefebvre A.-M., Saladin R., Najib J., Laville M., Fruchart J.-C., Deeb S. (1997). The Organization, Promoter Analysis, and Expression of the Human PPARγ Gene. J. Biol. Chem..

