# Supplementary material for: The Preservation of PPARγ Genome Duplicates in Some Teleost Lineages: Insights into Lipid Metabolism and Xenobiotic Exploitation
Source: Genes (Basel). 2022 Jan 4;13(1):107. doi: 10.3390/genes13010107 (PMC8774674; doi:10.3390/genes13010107)
Supplement: Supplementary file 1 [file genes-13-00107-s001.zip › genes-1511448-supplementary.pdf]

SUPPORTING INFORMATION

The preservation of PPARγ genome duplicates in some teleost lineages: insights into lipid metabolism and regulation

Inês Páscoa[a], Elza Fonseca[a], Renato Ferraz[a,b], André M. Machado[a], Francisca Conrado[a], Raquel Ruivo[a], Isabel Cunha[a*] and L. Filipe C. Castro[a,c*]

[a] CIIMAR/CIMAR—Interdisciplinary Centre of Marine and Environmental Research, University of Porto, 4450-208 Portugal

[b] ICBAS - Instituto de Ciências Biomédicas Abel Salazar, University of Porto, 4050-313 Portugal

[c] FCUP - Faculty of Sciences, Department of Biology, University of Porto, 4169-007 Portugal

Table S1: Accession numbers of the sequences used to perform the phylogenic analysis

Species name	Order	PPARα	PPARβ	PPARγ	
Homo sapiens	Primates	NP_005027.2	NP_001165289.1	NP_001341596.2	
Mus musculus	Rodentia	NP_035274.2	NP_035275.1	NP_001120802.1	
Anolis carolinensis	Squamata	XP_003221452.1	XP_003220387.1	XP_016847098.1	
Xenopus tropicalis	Anura	XP_002940784.2	XP_004910760.1	XP_031757160.1	
Gallus gallus	Galliformes	XP_015145415.1	NP_990059.2	NP_001001460.1	
Tetraodon nigroviridis	Tetraodontiformes			A-CAG07050.1	
Dentex dentex	Perciformes			A-ABO69005.1	
Epinephelus coioides	Perciformes			A-AJF19172.1	
Lateolabrax japonicus	Perciformes			A-ABC70398.1	
Lates calcarifer	Perciformes			A-XP_018542407.1	
Pagrus major	Perciformes			A-BAF80459.1	
Siniperca chuatsi	Perciformes			A-AMY62090.1	
Sparus aurata	Perciformes			A-AAT85618.1	
Poecilia formosa	Cyprinodontiformes			A-XP_007549555.1	
Xiphophorus maculatus	Cyprinodontiformes			A-XP_023181182.1	
Neolamprologus brichardi	Cichliformes			A-XP_006783314.1	
Oreochromis niloticus	Cichliformes			A-XP_025762757.1	
Pundamilia nyererei	Cichliformes			A-XP_005724711.1	
Paralichthys olivaceus	Pleuronectiformes			A-XP_019952399.1	
Pleuronectes platessa	Pleuronectiformes			A-CAD62449.1	
Seriola dumerili	Carangiformes			A-XP_022604094.1	

Esox lucius	Esociformes			A-XP_034153380.1	B-XP_010900626.1
Oncorhynchus kisutch	Salmoniformes			A-XP_020339632.1	B1-XP_020361547.1<br>B2-XP_020315616.2
Oncorhynchus mykiss	Salmoniformes			A-XP_021466520.1	B1-CDQ91583.1<br>B2-XP_021422381.1
Salmo salar	Salmoniformes			A-ENSSSAG00000029040.1	B1-XP_014000887.1<br>B2-XP_013990587.1
Ictalurus punctatus	Siluriformes			A-XP_017305851.1	B-XP_017343406.1
Tachysurus fulvidraco	Siluriformes			A-AGX15443.1	
Astyanax mexicanus	Characiformes			A-XP_007229695.2	B-XP_022532453.1
Pygocentrus nattereri	Characiformes			A-XP_017560263.1	B-XP_017553762.1
Ctenopharyngodon idella	Cypriniformes			A-ACF70732.1	
Cyprinus carpio	Cypriniformes			A-XP_018951590.1	
Danio rerio	Cypriniformes	NP_001096037.1	A-XP_699900.6<br>B-NP_571543.1	A-NP_571542.1	
Megalobrama amblycephala	Cypriniformes			A-ADH95692.2	
Misgurnus anguillicaudatus	Cypriniformes			A-AMR58838.1	
Sinocyclocheilus anshuiensis	Cypriniformes			A-XP_016340076.1	
Sinocyclocheilus rhinocerous	Cypriniformes			A-XP_016431122.1	
Clupea harengus	Clupeiformes			A-XP_012688561.1	B-XP_012682864.2
Sardina pilchardus	Clupeiformes			*	*
Scleropages formosus	Osteoglossiformes			A-KPP68409.1	
Scleropages formosus	Osteoglossiformes			A-KPP68409.1	
Lepisosteus oculatus	Lepisosteiformes	XP_015208771.1	XP_015198262.1	XP_006631094.2	

Legend: * - sequences isolated in the present work

Table S2: Relative gene expression levels of *PPARγA* and *PPARγB* in *Sardina pilchardus*. The values are presented in transcript per million (TPM) and log2 (TPM+1) ratios.

Gene	Metric	Spleen	Midgut	Red Muscle	Kidney	Liver	Gonad	Head Kidney	Gill and Branchial Arch	Caudal Fin (Skin + Cartilage + Bone)	Brain + Pituitary gland	White Muscle
**PPARγA**	TPM	2,90	85,25	3,96	8,89	12,68	4,80	10,95	16,31	6,29	24,40	0,89
	log2 (TPM+1)	1,96	6,43	2,31	3,31	3,77	2,54	3,58	4,11	2,87	4,67	0,92
**PPARγB**	TPM	0,00	17,02	0,00	2,27	8,11	0,00	3,70	0,00	0,00	0,05	0,32
	log2 (TPM+1)	0,00	4,17	0,00	1,71	3,19	0,00	2,23	0,00	0,00	0,07	0,40

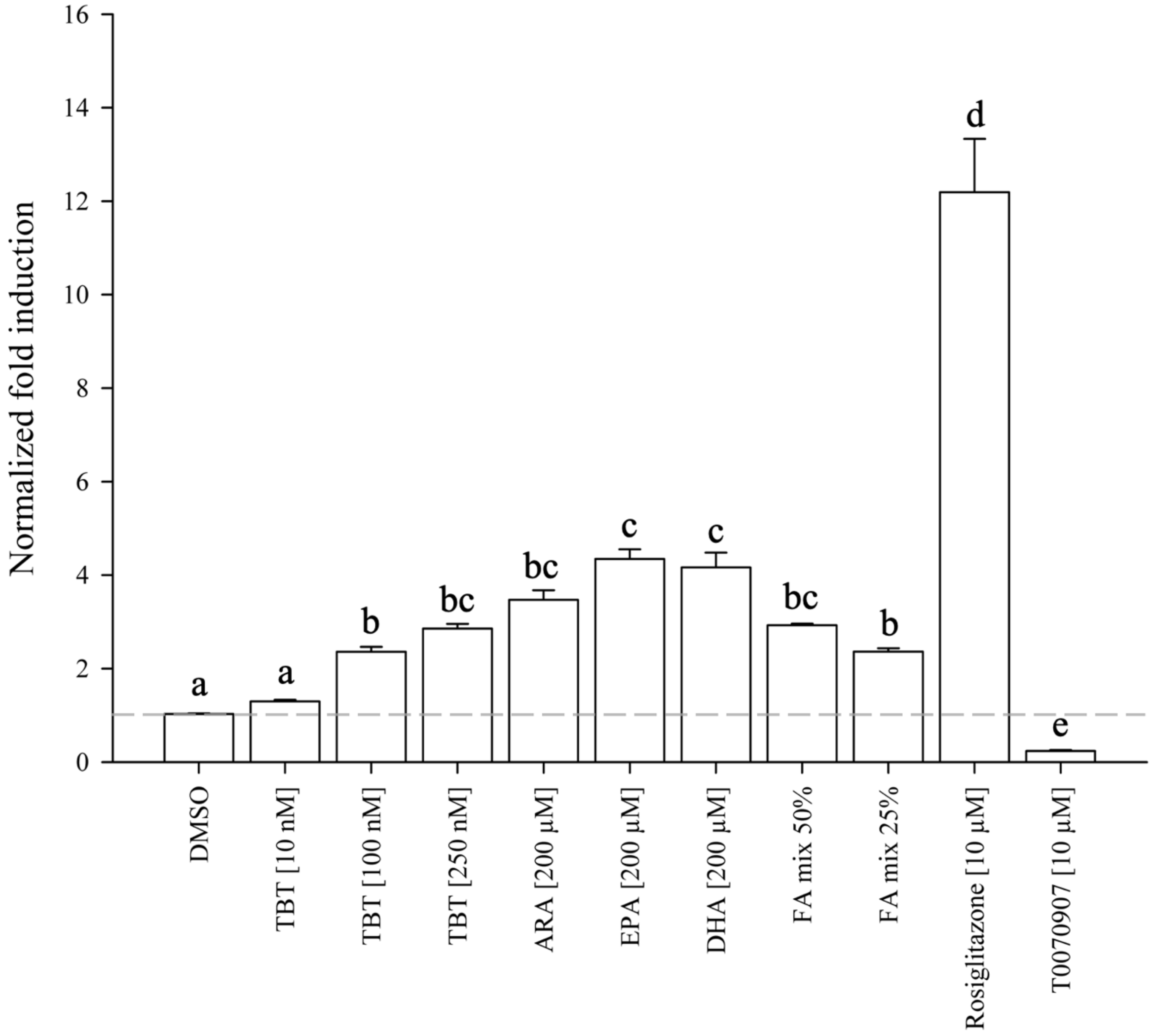

Figure S1: In vitro transcriptional activation of *Homo sapiens* PPARγ in the presence of three different concentrations of tributyltin chloride (TBT; 10 nM, 100 nM and 250 nM), two concentrations of a mixture of seven fatty acids (FA mix; 50% and 25%) and one concentration of arachidonic acid (ARA, 200 µM), eicosapentaenoic acid (EPA, 200 µM), docosahexaenoic acid (DHA, 200 µM), rosiglitazone (human PPARγ agonist; 10 µM) and T0070909 (human PPARγ antagonist; 10 µM). Dimethyl sulfoxide (DMSO; 0.1%) was used as solvent control. Data are shown as mean ± standard error of the mean (SEM).
